# Synergistic Benefits: Exploring the Anti-Virulence Effects of Metformin/Vildagliptin Antidiabetic Combination against *Pseudomonas aeruginosa* via Controlling Quorum Sensing Systems

**DOI:** 10.3390/biomedicines11051442

**Published:** 2023-05-14

**Authors:** Maan T. Khayat, Hisham A. Abbas, Tarek S. Ibrahim, Samar S. Elbaramawi, Ahdab N. Khayyat, Majed Alharbi, Wael A. H. Hegazy, Fatma Al-zahraa A. Yehia

**Affiliations:** 1 Department of Pharmaceutical Chemistry, Faculty of Pharmacy, King Abdulaziz University, Jeddah 21589, Saudi Arabia; tmabrahem@kau.edu.sa (T.S.I.); ankhayyat@kau.edu.sa (A.N.K.); maaalharbi1@kau.edu.sa (M.A.); 2 Department of Microbiology and Immunology, Faculty of Pharmacy, Zagazig University, Zagazig 44519, Egypt; hishamabbas2008@gmail.com (H.A.A.); zahra.ahmed.yehia@gmail.com (F.A.-z.A.Y.); 3 Medicinal Chemistry Department, Faculty of Pharmacy, Zagazig University, Zagazig 44519, Egypt; ssel-baramawy@pharmacy.zu.edu.eg; 4 Pharmacy Program, Department of Pharmaceutical Sciences, Oman College of Health Sciences, Muscat 113, Oman

**Keywords:** anti-virulence agents, quorum sensing, *Pseudomonas aeruginosa*, healthcare, vildagliptin, metformin

## Abstract

The repurposing of drugs is one of the most competent strategies for discovering new antimicrobial agents. Vildagliptin is a dipeptidyl peptidase-4 inhibitor (DPI-4) that is used effectively in combination with metformin to control blood glucose levels in diabetic patients. This study was designed to evaluate the anti-virulence activities of this combination against one of the most clinically important pathogens, *Pseudomonas aeruginosa*. The current findings show a significant ability of the vildagliptin–metformin combination to diminish biofilm formation, bacterial motility, and the production of virulent extracellular enzymes and pyocyanin pigment. Furthermore, this drug combination significantly increased the susceptibility of *P. aeruginosa* to oxidative stress, indicating immunity enhancement in the eradication of bacterial cells. In compliance with the in vitro findings, the histopathological photomicrographs of mice showed a considerable protective effect of the metformin–vildagliptin combination against *P. aeruginosa*, revealing relief of inflammation due to *P. aeruginosa*-induced pathogenesis. *P. aeruginosa* mainly employs quorum sensing (QS) systems to control the production of its huge arsenal of virulence factors. The anti-virulence activities of the metformin–vildagliptin combination can be interrupted by the anti-QS activities of both metformin and vildagliptin, as both exhibited a considerable affinity to QS receptors. Additionally, the metformin–vildagliptin combination significantly downregulated the expression of the main three QS-encoding genes in *P. aeruginosa*. These findings show the significant anti-virulence activities of metformin–vildagliptin at very low concentrations (10, 1.25 mg/mL, respectively) compared to the concentrations (850, 50 mg/mL, respectively) used to control diabetes.

## 1. Introduction

*Pseudomonas aeruginosa* is an aggressive pathogen responsible for severe infections in different body systems, including the urinary and respiratory tracts and the vascular and central nervous systems [1,2,3]. *P. aeruginosa* has been recently listed as a high-priority pathogen by the World Health Organization (WHO) [4,5,6], and it is considered as one of the most frequent etiologies of nosocomial infections and is associated with poor prognosis [7,8,9]. Naturally, *P. aeruginosa* has an intrinsic resistance to several antibiotics due to low outer membrane permeability [10,11]. Additionally, prolonged and recurrent exposure to bactericidal antibiotics in opportunistic pathogens leads to the emergence of antibiotic-resistant strains [9,12,13]. The establishment of bacterial infections has been related to biofilms, rugose small colony variants (RSCVs), hetero-resistance, and conventional antibiotic resistance [14,15]. Notably*, P. aeruginosa* had a wide range of antibiotic-tolerant and -resistant phenotypes at single-cell as well as population levels [16]. To overcome this encountered bacterial resistance, novel treatment tactics have to be introduced into treatment regimens [13,17]. Targeting bacterial virulence could be a possible option that disarms *P. aeruginosa* without exerting any selective pressure.

In order to cause infection, *P. aeruginosa* possesses a variety of virulence factors, which contribute to successful colonization, dissemination, and infection [18,19]. Pyocyanin is one of the most unique and important *P. aeruginosa* virulence factors that have a protective effect against harsh environmental conditions and an antimicrobial effect against competitors [20,21]. The redox-active molecule pyocyanin also contributes to biofilm formation, host tissue damage, and impaired organ function [5,22]. *P. aeruginosa* produces diverse extracellular enzymes including proteases, hemolysin, elastase, and lipase to establish its infection in the host tissues [8,11]. Additionally, *P. aeruginosa* exhibits many forms of motility, which have a crucial role in host colonization, dissemination, and host immune evasion [23,24]. Swarming motility in *P. aeruginosa* is a multicellular adaptation used for surface translocation that has been correlated with bacterial virulence and adaptive resistance to antibiotics [25,26]. 

Owing to the capacity of *P. aeruginosa* to form biofilm, it can successfully establish infections, including cystic fibrosis, chronic otitis media, chronic wound infection, and implanted medical device-associated infection, within susceptible hosts [27]. The sophisticated biofilm structure provides bacterial persistence against harsh environmental conditions, host defenses, and antimicrobial therapy [28,29]. The formation of biofilms is under the regulation of numerous mechanisms including quorum sensing (QS). Interestingly, QS systems in *P. aeruginosa* comprise four QS signaling mechanisms; two are Lux-type LasR/I, RhlR/I, and the orphan Lux-type analog IqsR which responds to diverse inducers, besides the particular pseudomonal non-Lux type PqsR which senses its own cognate produced by *pqs*A-D encoding genes [3,30,31]. That reflects the magnificent controlling QS systems orchestrating *P. aeruginosa* pathogenesis in different conditions by sensing diverse autoinducers, and in turn controlling a variety of genes implicated in bacterial virulence that ease the infection establishment and developing of antibiotic resistance [8,32,33,34,35,36]. In this context, there is a persistent demand to develop new approaches to control *P. aeruginosa* virulence and conquer its ability to develop resistance to antibiotics. 

In this direction, it is essential to find new antibiotics; however, the quick emergence of resistance within a short period could result in great economic, time, and effort loss. Alternatively, it was suggested to develop or even repurpose known drugs to serve as anti-virulence agents that could offer an efficient way to overcome bacterial resistance and financial hurdles [37,38,39,40,41]. Drug repurposing refers to the identification of new indications for existing drugs or the application of newly discovered mechanisms of action for known drugs [42,43]. The repurposing of old drugs for the treatment of antimicrobial-resistant pathogens has been explored as an alternative strategy in the field of antimicrobial drug discovery [44,45]. In the context of repurposing drugs for antimicrobial activity, there have been increasing reports of approved drugs being identified for this purpose [30,46,47,48,49]. Repositioning of non-antibiotic drugs as an alternative to antibiotics has become an attractive option due to the global spread of microbial resistance and the high costs and slow pace in the discovery of new antibiotics [45,50,51].

The main hypothesis of employing anti-virulence agents in diminishing bacterial resistance depends on the facts that (i) anti-virulence agents should not affect bacterial growth and hence do not cause bacteria to develop resistance [52,53], (ii) anti-virulence agents could attenuate the bacterial pathogenesis enabling the host immunity to neutralize the invading bacteria [53,54], and (iii) depending on the key role of QS systems in controlling bacterial virulence, anti-QS agents are proposed as efficient anti-virulence candidates [39,55,56]. Gliptins are a class of antidiabetic drugs that are dipeptidyl peptidase-4 inhibitors (DPI-4s) used for improving β-cell health and controlling blood glucose levels in diabetes mellitus type 2 [57]. In one of our leading studies, a molecular docking study was conducted to evaluate the gliptins’ binding affinities to *P. aeruginosa* and *Staphylococcus aureus*, and it was found that gliptins have anti-QS activities against these bacteria, showing in vitro and in vivo ability to diminish bacterial pathogenesis [3]. Metformin and vildagliptin are two commonly prescribed medications for the management of diabetes. Metformin, a biguanide, primarily works by reducing hepatic glucose production, improving insulin sensitivity, and decreasing intestinal glucose absorption [58]. On the other hand, vildagliptin, a DPP-4 inhibitor, enhances glycemic control by inhibiting the enzymatic degradation of incretin hormones, which play a crucial role in regulating glucose metabolism [3]. When used in combination, metformin and vildagliptin have demonstrated a synergistic effect in the treatment of diabetes. The combination therapy not only addresses the underlying insulin resistance but also targets the impaired incretin pathway. By leveraging the complementary mechanisms of action, the dual therapy offers improved glycemic control and a reduced risk of hypoglycemia compared to individual monotherapy options [59,60]. Additionally, studies have suggested that the metformin/vildagliptin combination may have additional benefits beyond glycemic control, such as potential cardiovascular protection and weight management [61]. Interestingly, metformin showed significant in vitro antibacterial [62,63,64] and anti-virulence activities [52,65]; however, it lacks in vivo effectiveness [52]. Vildagliptin is a cyanopyrrolidine-based hypoglycemic DPI-4 drug [57] that showed a considerable ability to hinder the QS receptors and downregulate their encoding genes [52,65]. Metformin is combined with vildagliptin as synergistic oral hypoglycemic tablets, in different concentrations [66]. The current study aimed to evaluate the anti-QS and anti-virulence efficacy of metformin and vildagliptin combinations against *P. aeruginosa*. 

## 2. Materials and Methods

### 2.1. Media and Chemicals 

Trypticase soya broth (TSB), Mueller Hinton (MH) broth and agar, Luria–Bertani (LB) broth, and trypticase soya agar (TSA) were obtained from Oxoid (Hampshire, UK). The chemicals, including dimethyl sulfoxide (DMSO), resazurin dye, crystal violet, and glacial acetic acid, were obtained from Sigma–Aldrich (St. Louis, MO, USA).

### 2.2. Bacterial Strain and Growth Condition 

*P. aeruginosa* (PAO1) was provided by the Department of Microbiology, Faculty of Pharmacy, Mansoura University. PAO1 was grown aerobically on trypticase soya agar at 37 °C. For long-term storage, PAO1 was maintained in Muller Hinton broth with glycerol (10–15%) and kept at −80 °C.

### 2.3. Detection of Minimum Inhibitory Concentrations (MICs) 

The broth microdilution method was employed to determine the MIC of metformin or vildagliptin separately or metformin and vildagliptin in combination against the PAO1 strain following the Clinical and Laboratory Standards Institute guidelines (CLSI, 2016) [38]. Briefly, 2-fold serial dilutions of vildagliptin or metformin were prepared in MH broth and added into a 96-well microtiter plate. PAO1 overnight culture in MH broth was diluted to an approximate cell density of 1 × 10^6^ CFU/mL. The adjusted PAO1 suspensions were added to wells with a final concentration of 5 × 10^5^ CFU/mL and incubated overnight at 37 °C. The MICs were considered as the lowest concentrations of metformin or vildagliptin that inhibited the growth of PAO1.

### 2.4. Determination of Effect of Sub-MICs of Metformin and Vildagliptin on PAO1 Growth and Metabolic Activity

The effect of metformin or vildagliptin on PAO1 growth and metabolic activity was evaluated by measuring the optical density of bacterial suspension and Alamar Blue assay, respectively. PAO1 was cultured in TSB broth containing metformin and vildagliptin at sub-MICs in addition to TSB broth as an untreated control. After incubation, bacterial suspension was measured at 600 nm [3,65]. 

A stock solution of resazurin dye was made in phosphate-buffered saline (PBS) (6.5 mg/mL). Treated cells as well as control cells were collected, washed twice, and then resuspended in PBS. Resazurin (100 µL) and cell suspension (900 µL) were added and incubated at 37 °C in the dark for 4 h. A blank of sterile PBS with resazurin was included. Then, the samples were centrifuged, and the fluorescence intensity of the supernatant containing the reduced resazurin was detected at 590/560 nm (emission/excitation) [67].

### 2.5. Phenotypic Characterization of P. aeruginosa Virulence

Sub-MICs of metformin or vildagliptin were concurrently used to assess their anti-virulence activity against PAO1. 

#### 2.5.1. Pyocyanin Assay

The effect of metformin or vildagliptin at sub-MICs on pyocyanin biosynthesis was determined as described previously [68,69]. An overnight culture of PAO1 in TSB broth was diluted to an OD600 of 0.4. The diluted suspension (10 μL) was inoculated in 1 mL of LB broth in the presence and absence of sub-MICs of metformin or vildagliptin separately or metformin and vildagliptin in combination. After 48 h incubation at 37 °C, the cultures were centrifuged, and the absorbance of pyocyanin in the supernatants was measured at 691 nm. 

#### 2.5.2. Oxidative Stress Resistance Assay 

The effect of metformin or vildagliptin separately or metformin and vildagliptin in combination at sub-MICs on pyocyanin-mediated resistance to oxidative stress was evaluated by the cup diffusion method [52,70]. An overnight culture of PAO1 (100 µL) was uniformly spread on the surface of TSA plates supplemented with sub-MICs of metformin and/or vildagliptin. Cups were made into agar plates and filled with 20 µL of hydrogen peroxide (1.5%). The plates were overnight incubated aerobically at 37 °C, and the diameters of the inhibition zones were measured in mm. 

#### 2.5.3. Skim Milk Broth Assay for Total Protease Activity

The effect of metformin or vildagliptin separately or metformin and vildagliptin in combination at sub-MICs on PAO1 total protease production was assessed using a modified skimmed milk broth method [71,72]. PAO1 was grown overnight in MH broth in the presence of sub-MICs of metformin and/or vildagliptin. Then, the bacterial suspensions were centrifuged, and the supernatants (500 µL) were added to 1.25% skimmed milk (1 mL) at 37 °C for 1 h. The optical density of skimmed milk was measured at 600 nm and compared to untreated control. 

#### 2.5.4. Biofilm Inhibition Assay 

The PAO1 ability to form biofilms in the presence of sub-MICs of metformin or vildagliptin separately or metformin and vildagliptin in combination was examined by employing the modified method of Stepanovic et al. [73,74]. Briefly, suspensions of PAO1 overnight growth were adjusted to 1 × 10^6^ CFU/mL and transferred in aliquots of 100 μL to the wells of 96-well sterile microtiter plates, to be incubated for 24 h at 37 °C. Then the planktonic cells were aspirated and the wells were washed 3 times with sterile water. The formed biofilms were fixed with aliquots of 100 μL of 99% methanol for 20 min. Then, the wells were stained for 20 min with crystal violet (1%), and the excess dye was washed out. After drying in air, the dye was eluted by the glacial acetic acid (33%), and the absorbances were measured at 590 nm. 

#### 2.5.5. Motility Inhibition Assay 

The influence of metformin or vildagliptin separately or metformin and vildagliptin in combination at sub-MICs on swarming motility was investigated as described previously [48,75]. Prior to use, MH agar plates were dried overnight at room temperature. Two-microliter overnight cultures of untreated and treated PAO1 were spotted on the surface of swarming agar plates. After incubation at 37 °C for 24 h, the diameters of swarming motility were measured, and plates were photographed.

### 2.6. Quantitative RT-PCR of QS-Encoding Genes

Cell pellets of overnight culture of control and treated PAO1 were obtained by centrifugation. A TRIzol RNA extraction and purification kit (Life Technologies, Carlsbad, CA, USA) was used according to manufacturer protocol. Extracted RNA was evaluated using a NanoDrop (ND-1000 spectrophotometer) (Wilmington, DE, USA) at 260 nm and 280 nm to ensure the quality of RNA yield and saved at −80 °C [76,77].

In this study, the expression levels of the QS-encoding genes were determined using the comparative threshold cycle (∆∆Ct) method, which was described in previous literature [76,78]. To standardize the expression levels, the housekeeping gene *ropD* was used as a reference. The cDNA was synthesized using the high-capacity cDNA reverse transcriptase kit from Applied Biosystem (Waltham, MA, USA), and amplification was performed using the Syber Green I PCR Master Kit from Fermentas (Waltham, MA, USA). The Step One instrument from Applied Biosystem was used for the amplification process. The primers utilized in this study are listed in [2,47,69,79].

### 2.7. Histopathological Evaluation of the Protective Effect of Metformin–Vildagliptin against PAO1

In order to assess the anti-virulence activity of the combination of metformin and vildagliptin in vivo, sub-MIC intra-peritoneal injections of metformin and vildagliptin were administered to three-week-old Mus musculus (albino mice), and histopathological examination of kidney and liver tissues was carried out, following methods previously described in the literature [38,47,72]. The mice were divided into five groups of five individuals each. The first group received intra-peritoneal injections of metformin and vildagliptin combination-treated PAO1 (1 × 10^6^ CFU/mL) as a test group. The second and third groups received intra-peritoneal injections of untreated PAO1 (1 × 10^6^ CFU/mL) or DMSO-treated PAO1, serving as positive control groups. The fourth and fifth groups were either injected with sterile PBS or kept un-injected to serve as negative control groups. After a five-day observation period, the mice were euthanized by cervical dislocation, and their livers and kidneys were removed and rinsed with normal saline. The tissues were then fixed in neutral buffered formalin (10%). To prepare the tissues for histopathological examination, the samples were dehydrated with increasing concentrations of ethanol (70%, 90%, and 100%) and cleared in xylol. The tissues were then embedded in paraffin wax, and 5μm thick sections were cut using a rotatory microtome. The sections were stained with hematoxylin and eosin (H&E) for observation under a light microscope.

### 2.8. Virtual Study to Evaluate Metformin and Vildagliptin Affinity to Bind to QS Receptors 

*P. aeruginosa* crystal structures of LasR (PDB code: 1RO5/ 2.30 Å) [80], QscR (PDB code: 6CC0/ 2.50 Å) [81], and PqsR (PDB code: 6MVN/ 2.20 Å) [82] were retrieved from the RCSB Protein Data Bank (https://www.rcsb.org/, accessed on 25 September 2022) [69]. The receptor structures were prepared by following the QuickPrep protocol on Molecular Operating Environment (MOE 2019.012) with Amber10: EHT forcefield [31]. Vildagliptin and metformin were obtained from the PubChem database (https://pubchem.ncbi.nlm.nih.gov/, accessed on 18 September 2022) as canonical SMILES. Each drug structure was prepared individually through energy minimization using 0.1 Kcal/mol/Å² gradient RMS, followed by protonate 3D at physiological pH 7.4. Docking procedures were performed through Alpha triangle placement with Amber10: EHT forcefield.

### 2.9. Statistical Analysis 

The experiments were carried out in triplicate, and the results are presented as the mean ± SD. The statistical significance of the inhibitory activities was evaluated using unpaired one-way ANOVA followed by Dunnett posttest in Graph Pad Prism 8, and *p* values below 0.05 were considered to be statistically significant.

## 3. Results

### 3.1. Determination of Metformin and/or Vildagliptin MIC Values against PAO1

The minimum concentrations of metformin and vildagliptin that inhibited visible PAO1 growth were 100 and 20 mg/mL, respectively. The sub-MICs of metformin and vildagliptin (10 and 1.25 mg/mL, respectively) were used in combination to assess their inhibitory activities on PAO1 virulence in the former experiments.

### 3.2. Metformin and/or Vildagliptin at Sub-MICs Did Not Affect Bacterial Growth or Metabolic Activity

**Figure 1:**
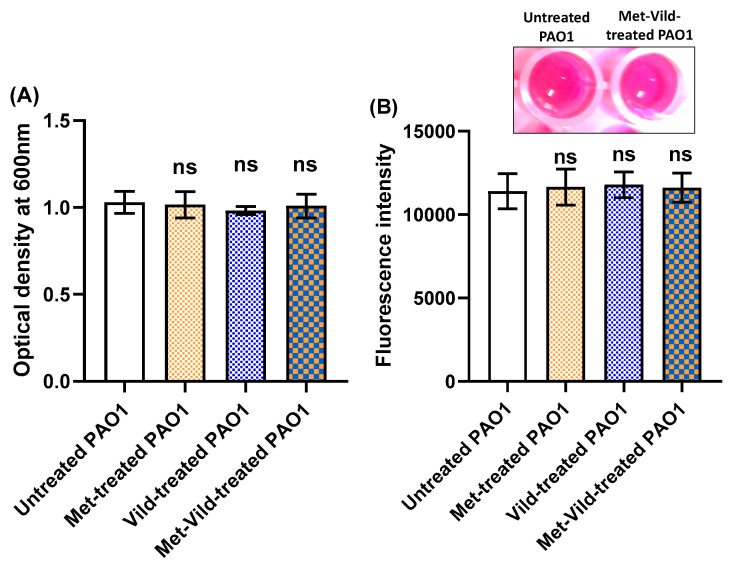
Sub-MICs of metformin and/or vildagliptin did not affect PAO1 growth or metabolic activity. (**A**) The effect of sub-MICs of metformin and vildagliptin on PAO1 growth as indicated by bacterial optical density at 600 nm. (**B**) The effect of sub-MICs of metformin and vildagliptin on PAO1 metabolic activity as indicated by Alamar Blue assay (insert photograph represents reduced resazurin dye in untreated and treated PAO1 cells on the left and right, respectively). Data shown represent the mean ± standard error from triplicate experiments (ns: non-significant).

Sub-MICs of metformin and vildagliptin did not affect *P. aeruginosa* PAO1 growth, as indicated by optical densities of overnight cultures grown in the presence or absence of metformin or vildagliptin separately or metformin and vildagliptin in combination at sub-MICs (Figure 1A). Moreover, the Alamar Blue assay was performed and showed no significant difference in the metabolic activity of metformin- and/or vildagliptin-treated cells compared to untreated cells (Figure 1B). These results indicate that sub-MICs of metformin and/or vildagliptin have no effect on either bacterial growth or metabolic activity. These results could exclude that the anti-virulence activities of metformin and vildagliptin are due to PAO1 inhibition of growth.

### 3.3. Metformin and Vildagliptin Combination at Sub-MICs Reduced Pyocyanin Production 

**Figure 2:**
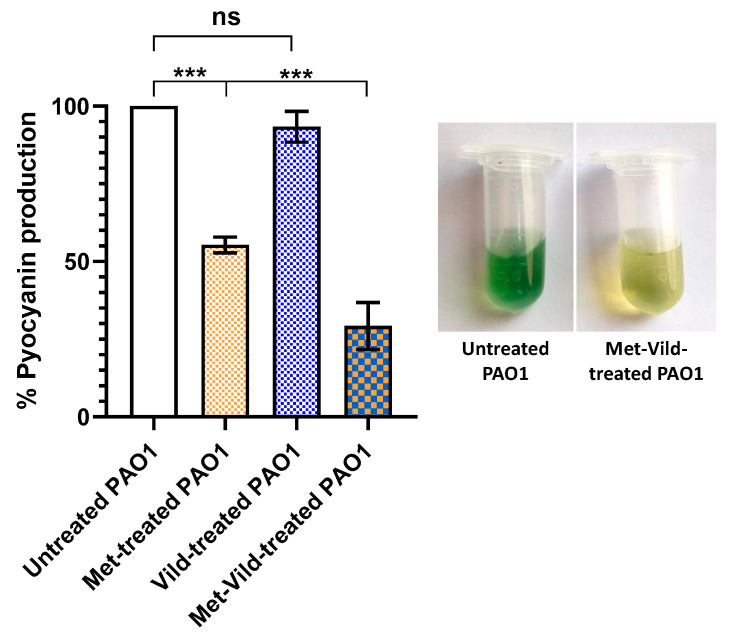
Sub-MICs of metformin and vildagliptin significantly reduced pyocyanin biosynthesis in PAO1. Pyocyanin production was significantly inhibited in treated PAO1 cells compared to untreated control and only metformin- or vildagliptin-treated bacterial cells. Data shown represent the mean ± standard error from three experiments (***: *p* value < 0.001; ns: non-significant).

The impact of the sub-MICs of metformin and vildagliptin on pyocyanin biosynthesis was spectrophotometrically estimated. While vildagliptin at sub-MIC did not show a significant effect on pyocyanin production, metformin at sub-MIC showed a significant inhibitory effect. Cells treated with metformin and vildagliptin exhibited significantly reduced pyocyanin production (29.3% ± 3.1) compared to untreated cells and to only metformin- or vildagliptin-treated bacterial cells (Figure 2). 

### 3.4. Metformin and Vildagliptin at Sub-MICs Sensitized PAO1 to Oxidative Stress

**Figure 3:**
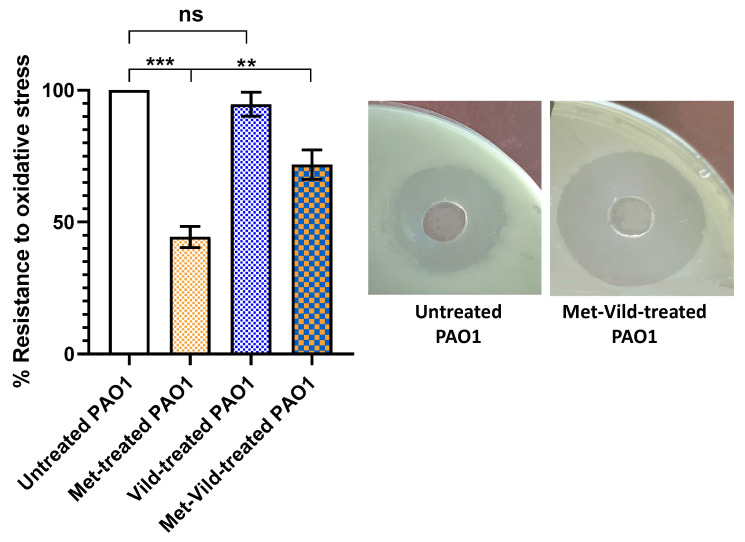
Sub-MICs of metformin and vildagliptin sensitized PAO1 to oxidative stress. Increased PAO1 susceptibility to hydrogen peroxide upon treatment with sub-MICs of metformin and vildagliptin compared to untreated control. The cup diffusion method showed an increased diameter of the hydrogen peroxide inhibition zone in treated PAO1 cells. Metformin at sub-MIC showed a significant inhibitory effect on oxidative stress, while vildagliptin at sub-MIC did not show a significant effect. Data shown represent the mean ± standard error from three experiments (**: *p* value < 0.01; ***: *p* value < 0.001; ns: non-significant).

The effect of metformin and vildagliptin on pyocyanin-mediated oxidative stress was assessed by hydrogen peroxide susceptibility assay. Bacteria treated with metformin and vildagliptin in combination at sub-MICs showed a significant reduction in resistance to oxidative stress (71.8% ± 3.2%) compared to control untreated cells, as indicated by the diameter of the hydrogen peroxide inhibition zone (Figure 3). It is worth mentioning that metformin at sub-MIC showed a significant reduction in oxidative stress, in contrast to vildagliptin which had no effect at sub-MIC.

### 3.5. Metformin and Vildagliptin at Sub-MICs Reduced Total Protease Production 

**Figure 4:**
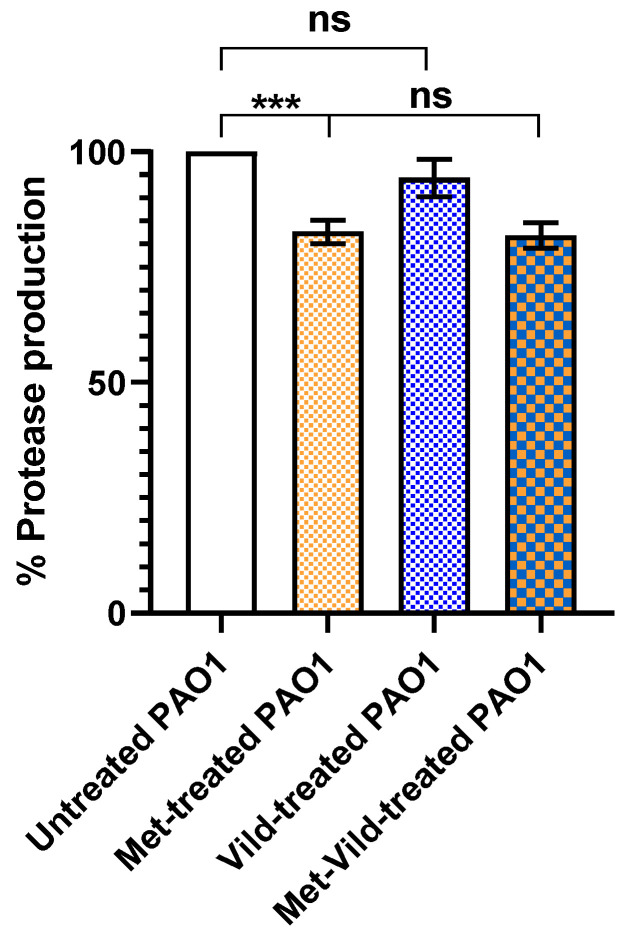
Sub-MICs of metformin and vildagliptin reduced PAO1 total proteolytic activity. Data shown represent the mean ± standard error from three experiments (***: *p* value < 0.001; ns: nonsignificant). Vildagliptin at sub-MIC had no significant influence, while metformin showed a significant inhibitory effect on protease reduction.

The effect of metformin and vildagliptin on PAO1 proteolytic activity was estimated using the modified skimmed milk broth assay method. Bacteria treated with the combination of metformin and vildagliptin at sub-MICs exhibited significantly lower skim milk proteolysis (81.9% ± 1.6%) compared to untreated PAO1 (Figure 4). Vildagliptin at sub-MIC had no significant effect on protease production, while metformin at sub-MIC significantly reduced the production of proteases. 

### 3.6. Metformin and Vildagliptin at Sub-MICs Inhibited Biofilm Formation

**Figure 5:**
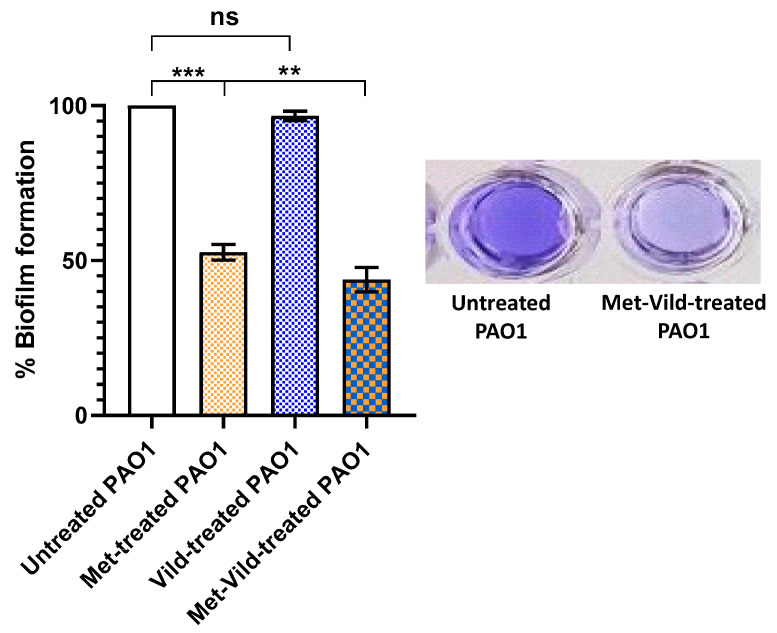
Sub-MICs of metformin and vildagliptin inhibited PAO1 biofilm formation, as evaluated by crystal violet quantification assay. While metformin at sub-MIC significantly inhibited biofilm formation, vildagliptin had no significant effect. Data shown represent the mean ± standard error from three experiments (**: *p* value < 0.01; ***: *p* value < 0.001; ns: non-significant).

The effect of metformin and vildagliptin on PAO1 biofilm formation was assessed by the crystal violet quantification method. Bacterial cells treated with metformin and vildagliptin at sub-MICs exhibited a significantly reduced biofilm-forming capability (43.8 % ± 1.6%) compared to control untreated cells or cells treated with only metformin or vildagliptin at sub-MICs (Figure 5). Vildagliptin at sub-MIC had no significant influence on biofilm formation, while metformin at sub-MIC significantly inhibited the formation of bacterial biofilm.

### 3.7. Metformin and Vildagliptin at Sub-MICs Decreased PAO1 Swarming Motility 

**Figure 6:**
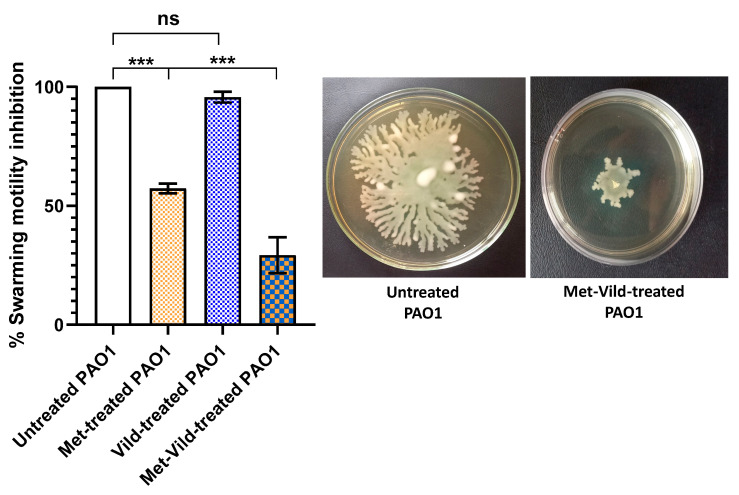
Sub-MICs of metformin and vildagliptin inhibited PAO1 swarming motility. Significant decrease in PAO1 swarming motility of treated cells compared to control untreated bacteria or to cells treated with only metformin or vildagliptin at sub-MICs. Data shown represent the mean ± standard error from three experiments (***: *p* value < 0.001; ns: non-significant).

The effect of metformin and vildagliptin on PAO1 swarming motility was evaluated. Treated cells showed decreased capacities to swarm on an agar surface (18.27% ± 1.5) compared to control untreated cells or cells treated with only metformin at sub-MIC or vildagliptin at sub-MIC (Figure 6). Vildagliptin at sub-MIC had no significant influence on bacterial swarming motility, while metformin at sub-MIC showed a significant inhibitory effect. 

### 3.8. Metformin and Vildagliptin at Sub-MICs Altered PAO1 QS Genes’ Expression

**Figure 7:**
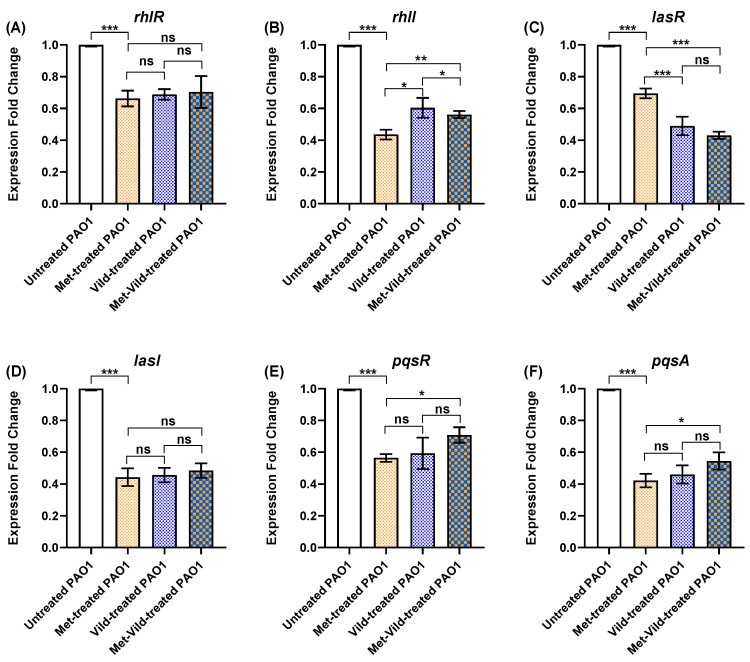
Sub-MICs of metformin and vildagliptin altered PAO1 QS gene expression. RT-qPCR revealed decreased expression of QS-encoding genes (**A**) rhlR, (**B**) rhlI, (**C**) lasR, (**D**) lasI, (**E**) pqsR, and (**F**) pqsA in treated PAO1 cells compared to control untreated bacteria. Data shown represent the mean ± standard error from three experiments (*: *p* value ≤ 0.05; **: *p* value < 0.01; ***: *p* value < 0.001; ns: non-significant).

The influence of metformin and vildagliptin treatment on the expression of PAO1 QS-encoding genes was evaluated by quantitative real-time PCR. The expression levels of rhlR, rhlI, lasR, lasI, pqsA, and pqsR were significantly decreased after PAO1 treatment with sub-MICs of metformin or vildagliptin separately or metformin and vildagliptin in combination compared to the untreated control (Figure 7). 

### 3.9. Metformin and Vildagliptin Show Virtual Affinity to the Main Three Pseudomonas QS Receptors 

**Figure 8:**
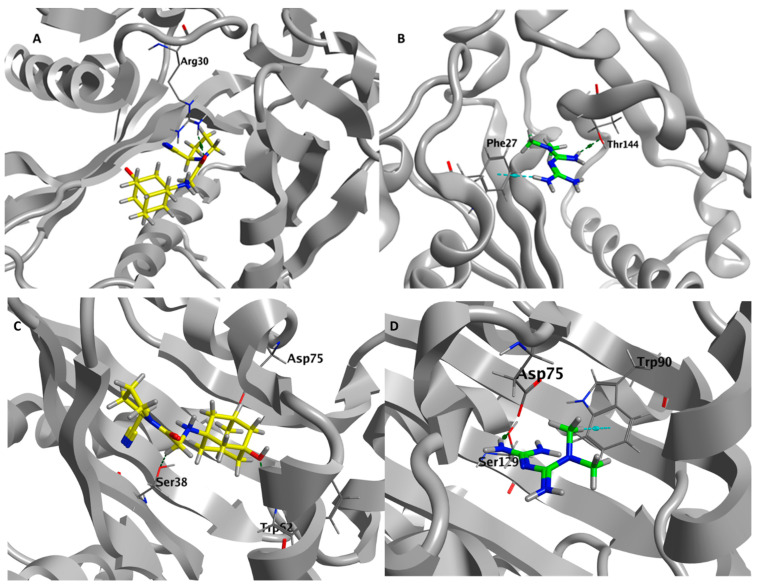

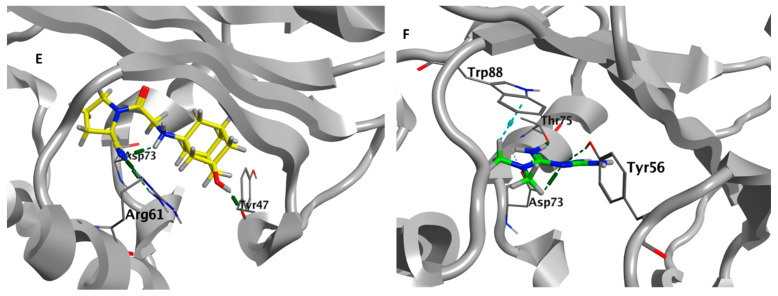
(**A**) Three-dimensional vildagliptin–*P. aeruginosa* LasR (PDB code: 1RO5); (**B**) 3D metformin–*P. aeruginosa* LasR (PDB code: 1RO5); (**C**) 3D vildagliptin–*P. aeruginosa* QscR (PDB code: 6CC0); (**D**) 3D metformin–*P. aeruginosa* QscR (PDB code: 6CC0); (**E**) 3D vildagliptin–*P. aeruginosa* pqsR (PDB code: 6MVN) interaction diagram; (**F**) 3D metformin–*P. aeruginosa* PqsR (PDB code: 6MVN) interaction diagram. Vildagliptin and metformin are thick yellow and green sticks, respectively. H-bonds and H-arene bonds are shown as green and cyan dots, respectively.

Molecular docking was performed to gain insights into the molecular interactions of vildagliptin and metformin on *P. aeruginosa* quorum sensing receptors. There is no co-crystallized ligand for *P. aeruginosa* LasR (PDB code: 1RO5), so the MOE site finder module was utilized for active pocket prediction. Vildagliptin and metformin showed good binding energy scores (S score = −5.8358 and −4.6581 Kcal/mol, respectively). The Carbonyl group of vildagliptin exhibited H-bond interaction with the basic Arg30. The protonated amino group of metformin formed a H-bond with Thr144, and one of the terminal amino groups showed H-arene interaction with Phe27. For *P. aeruginosa* QscR (PDB code: 6CC0), the co-crystalized ligand showed a docking energy score of −10.1568 Kcal/mol. The protonated amino group of both vildagliptin and metformin exhibited ionic bond interaction with the acidic Asp75. Moreover, vildagliptin showed H-bonds with Trp62 and Ser38. Metformin formed a H-bond with Ser129 and H-arene interaction with Trp90. Binding energy scores of vildagliptin and metformin are promising: −7.2594 and −5.2485 Kcal/mol, respectively. For *P. aeruginosa* pqsR (PDB code: 6MVN), the co-crystalized ligand showed a docking energy score of −9.1325 Kcal/mol. Vildagliptin formed H-bonds with Tyr47, Arg61, and Asp73 with a binding energy score of −6.0766 Kcal/mol. Metformin formed H-bond interaction with Tyr56, Thr75, and Asp73 and formed H-arene interaction with Trp88 with a binding energy score of −5.4563 Kcal/mol. The simultaneous interactions of vildagliptin and metformin on *P. aeruginosa* LasR, QscR, and PqsR are described in Figure 8.

### 3.10. Metformin and Vildagliptin Diminish the P. Aeruginosa Pathogenesis 

**Figure 9:**
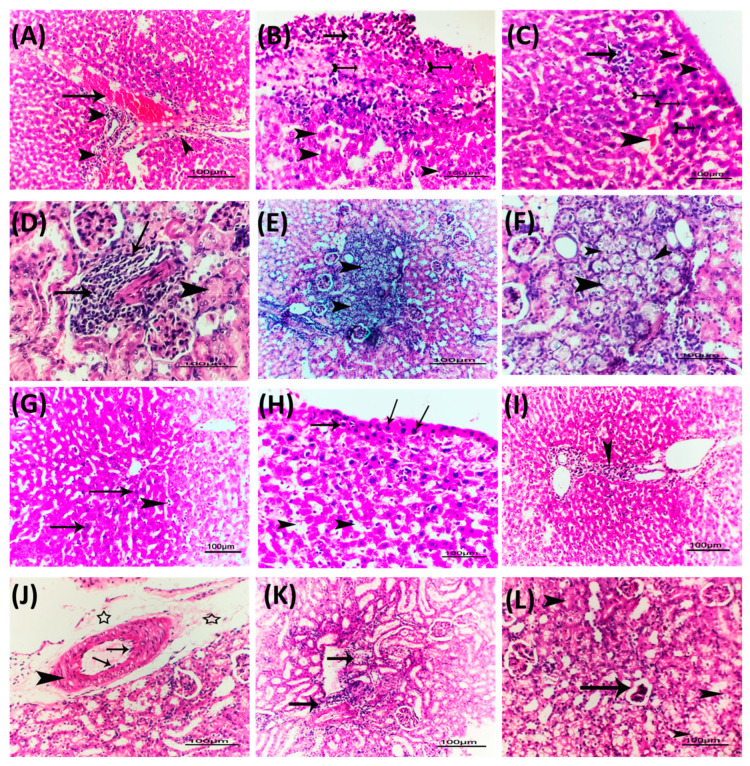
Metformin and vildagliptin diminished the *P. aeruginosa* pathogenesis. The mice were divided into 5 groups of five female, three-week-old mice each. The test group was intra-peritoneally injected with metformin and vildagliptin combination-treated PAO1 (1 × 10^6^ CFU/mL), and there were two negative control groups (un-injected and PBS-injected) and two positive groups (injected with untreated PAO1 or DMSO-treated PAO1). After observation for 5 days, the mice were euthanized, and their livers and kidneys were isolated for examination. Histopathological photomicrographs of the liver and kidney tissues (H&E × 200) from infected mice with untreated *P. aeruginosa* or with metformin and vildagliptin combination-treated *P. aeruginosa* mice groups were taken. (**A**) Photomicrograph of H&E-stained liver section of group infected with untreated PAO1 showing severe congestion of hepatic blood vessel (arrow) with perivascular inflammatory cell infiltration (arrowhead). (**B**) Photomicrograph of H&E-stained liver section of group infected with untreated PAO1 showing caseous necrosis (tailed arrows) with perivascular inflammatory cell infiltration (arrow) and individualization of some hepatocytes. (**C**) Photomicrograph of H&E-stained liver section of group infected with untreated PAO1 showing subcapsular congestion of both blood sinusoids and blood vessels (arrowhead) with nuclear hyperchromasia of some hepatocytes (tailed arrow) and focal leucocytic cellular proliferation (arrow). (**D**) Photomicrograph of H&E-stained kidney section of group infected with untreated PAO1 showing focal leucocytic cellular proliferation (arrows) with degeneration of some renal tubules represented by cloudy swelling (arrowhead) in renal cortex. (**E**) Photomicrograph of H&E-stained kidney section of group infected with untreated PAO1 showing vacuolation of renal epithelium tubules of some renal tubules (arrowhead) in renal cortex. (**F**) High power of the previously demonstrated photomicrograph to show vacuolation of renal epithelium tubules of some renal tubules (arrowhead) in renal cortex. (**G**) Photomicrograph of H&E-stained liver section of group infected with tested combination-treated PAO1 showing apparently normal hepatic parenchyma architecture with mild hepatic nuclear hyperchromasia (arrows) and sinusoidal congestion (arrowhead). (**H**) Photomicrograph of H&E-stained liver section of group infected with tested combination-treated PAO1 showing mild subcapsular hepatic nuclear hyperchromasia (arrows) and dilation of hepatic sinusoids (arrowhead). (**I**) Photomicrograph of H&E-stained liver section of group infected with tested combination-treated PAO1 showing mild perivascular leucocytic cell infiltration (arrowhead). (**J**) Photomicrograph of H&E-stained kidney section of group infected with tested combination-treated PAO1 showing mild endotheliosis (arrows) with increased thickness of blood vessels (arrowhead) with perivascular edema (stars). (**K**) Photomicrograph of H&E-stained kidney section of group infected with tested combination-treated PAO1 showing intertubular leucocytic cell infiltration (arrows). (**L**) Photomicrograph of kidney section of group infected with tested combination-treated PAO1 showing fewer focal areas of cellular infiltration (arrows) (bar = 100 µm).

Representative photomicrographs were taken of the renal and liver tissues of mice infected with *P. aeruginosa* and treated with the combination of metformin and vildagliptin at sub-MICs to demonstrate its effectiveness in reducing *P. aeruginosa*-induced pathogenesis. The liver tissues isolated from mice injected with untreated *P. aeruginosa* exhibited severe congestion of liver blood vessels, perivascular fibrosis, and hydropic degeneration of hepatocytes (Figure 9A–C), as well as degenerative changes, swelling, and areas of cellular proliferation in renal tubules and caseous necrosis in kidney tissues (Figure 9D–F). On the other hand, mice injected with *P. aeruginosa* treated with metformin and vildagliptin showed only mild infiltration of von Kupffer cells, vacuolation of few hepatocytes, and mild congestion in hepatic blood vessels (Figure 9G–I). Furthermore, metformin and vildagliptin reduced *P. aeruginosa* pathogenesis in kidney tissues, where mild diffuse cystic dilation of renal tubules, fewer focal areas of cellular infiltration, and normal renal cortex were observed (Figure 9J–L). These results indicate that the combination comprising metformin and vildagliptin at sub-MICs has a beneficial effect in reducing *P. aeruginosa*-induced pathogenesis.

## 4. Discussion

*P. aeruginosa* is one of the most notable human pathogens; it uses a variety of competitive and cooperative strategies to thrive in different environments, using numerous virulence factors [83,84,85]. *P. aeruginosa* possesses most known antimicrobial resistance mechanisms, which is why common empirical antibiotic treatments are expected to be ineffective in most cases [86,87]. Therefore, novel therapeutic approaches are required to develop new antimicrobials [31,88]. Targeting the QS systems has advantages as it avoids directly affecting bacterial growth and decreases the emergence of resistance [89,90]. The current work aimed to test the anti-QS activities of metformin and vildagliptin in combination against *P. aeruginosa.*

Metformin and vildagliptin are used in combination to control diabetes type II at concentrations of 500/50 mg/mL [66]. The MICs of metformin or vildagliptin against *P. aeruginosa* were low (100 and 20 mg/mL, respectively) as compared to the concentrations used to control hyperglycemia. The main concept of targeting bacterial QS is attenuating the virulence without influencing the bacterial growth [52,91]; thus, the anti-QS and anti-virulence activities of metformin and/or vildagliptin were assessed at sub-MICs. The selected sub-MICs were 10 and 1.25 mg/mL for metformin and vildagliptin, respectively; these concentrations keep the same ratio of metformin to vildagliptin (almost 10:1) as that used in the hypoglycemic tablets. There was no effect of metformin or vildagliptin separately or metformin and vildagliptin in combination at sub-MICs on bacterial growth. 

*P. aeruginosa* possesses three main QS systems that control its virulence: two LuxI/LuxR QS systems and a non-LuxI/LuxR QS system called the PQS system [89]. LasI and RhlI synthases synthesize autoinducers C12-homoserine lactone and butanoyl homoserine lactone, respectively, to be sensed by QS receptors LasR and RhlR, respectively [92,93]. It is worth mentioning that QscR is another LuxR homolog that senses the autoinducers made by LasI [94]. Additionally, there is another non-Lux type QS receptor, namely PQS, which senses the autoinducers that are encoded on *pqsA-D* genes [95]. By the binding of autoinducers to their cognate receptors, they are able to interact with short DNA sequences of the bacterial chromosome such as Lux boxes controlling the expression of the downstream virulence genes [30,46,96]. Surprisingly, metformin or vildagliptin separately or metformin and vildagliptin in combination at sub-MICs significantly downregulated the expression of the three QS-encoding genes. Moreover, metformin in combination with vildagliptin lowered the expression of LasI/R-encoding genes as compared to single drugs. A docking study was conducted to evaluate the affinity of metformin and vildagliptin to the three main *P. aeruginosa* QS receptors, LasR, RhlR, and PqsR. The results showed a considerable affinity of metformin or vildagliptin to bind and interfere with the QS receptors. Based on these findings, it is supposed that metformin and vildagliptin possess anti-QS activities. 

The roles of QS in controlling bacterial virulence have been extensively studied and reviewed [90,97,98,99,100]. There is an important relation between biofilm formation and bacterial motility, it was found that non-motile bacterial mutants could lack the ability to form biofilms and that bacterial mutants lacking the ability to form biofilms could be non-motile [23,101,102,103,104]. The role of QS in the regulation of biofilm formation and bacterial motility is well studied [55,88,105,106,107]; intriguingly, metformin combined with vildagliptin at sub-MICs significantly diminished both biofilm formation and swarming motility. QS systems control a wide array of virulence factors in *P. aeruginosa*, including the production of extracellular enzymes such as protease, elastase, and hemolysin besides the characteristic *P. aeruginosa* bluish-green pigment pyocyanin [108,109]. The metformin and vildagliptin combination at sub-MICs significantly diminished the production of protease and pyocyanin in *P. aeruginosa*. Pyocyanin is known to kill competing microbes and mammalian cells through oxidation and reduction reactions [110]. In compliance with the significant effect of the metformin and vildagliptin combination on reduction in pyocyanin, the metformin and vildagliptin combination significantly reduced *P. aeruginosa*′s tolerance to oxidative stress. In complete agreement with the significant effects of the metformin and vildagliptin combination on reduction in virulence phenotypically, the in vivo results emphasized these findings. Histopathological photomicrographs of kidney and liver tissues of injected mice revealed the alleviation of *P. aeruginosa*-induced pathogenesis.

In previous studies, the anti-QS and anti-virulence activities of metformin and vildagliptin at sub-MIC, separately, were evaluated against *P. aeruginosa* [52] and *Serratia marcescens* [65]. In agreement with the current results, metformin at sub-MIC acquired a significant in vitro diminishing effect on the virulence of both *P. aeruginosa* and *S. marcescens*, while it lacks any in vivo activity. Vildagliptin lacks both in vivo and in vitro anti-virulence activities against *P. aeruginosa* and *S. marcescens*; however, it downregulated the expression of QS-encoding genes in *P. aeruginosa* [52] and virulence-encoding genes in *S. marcescens* [65]. Furthermore, vildagliptin and metformin showed affinity to bind to the QS receptors LasR, QscR, and PqsR in *P. aeruginosa* [52] and SmaR in *S. marcescens* [65]; however, the docking scores of metformin were higher than those of vildagliptin. The low docking score of vildagliptin was attributed to the planar nature of its bulky aliphatic adamantyl group, besides its rapid conformational changes which could be a barrier for fitting on QS receptors efficiently, which could explain the in vitro and in vivo inactivity [52]. On the other hand, metformin with its small very active biguanide moiety does not fit all active pockets on the QS receptors, but it is able to fit and sufficiently block QS receptors in a way that could result in significant anti-QS activities in vitro. The metformin activity is due to the non-ionized form that is very sensitive to acidic pH and rendered in an ionized inactive form, which may explain the in vivo inactivity due to a decrease in pH during microbial growth [111] that in turn inactivates metformin. 

In the current study, the metformin and vildagliptin combination significantly diminished the virulence of *P. aeruginosa* in vitro and showed obvious mitigation of its pathogenesis in vivo. Metformin was proven to be an efficient efflux pump inhibitor [112,113] that could increase the internalization of vildagliptin in bacterial cells. Taking into consideration the downregulation effects of vildagliptin and metformin on the expression of QS-encoding genes, the increased internalized vildagliptin could bind to and hinder the cytosolic QS receptors [92], which could explain the activity of the metformin and vildagliptin combination in vivo. Another consideration is the basic nature of vildagliptin [114], which could raise the pH to keep the unionized active form of metformin during bacterial growth, which also may enhance the in vivo activity of the combination. 

In summary, the metformin and vildagliptin combination showed significant anti-virulence activities in vitro and in vivo in very low concentrations (10/1.25 mg/mL). Bearing in mind that metformin and vildagliptin are used together as an antidiabetic in concentrations of 500/50 or 500/100, it is advisable to prescribe this combination to control bacterial infections besides its main purpose of use as an antidiabetic. Furthermore, these findings give the chance to use this combination in very low doses to be tested as an anti-virulence and antibiotic adjuvant without an effect on blood glucose levels; however, this requires further pharmacological assessment to determine the effective dose and exclude any side effects. 

## 5. Conclusions

The strategy of repurposing drugs is a highly effective approach to discovering new antimicrobial agents. This study was conducted to investigate the anti-virulence properties of the antidiabetic metformin–vildagliptin combination at sub-MICs against *P. aeruginosa*. The results reveal that the vildagliptin–metformin combination considerably reduces biofilm formation, bacterial motility, and the production of virulent extracellular enzymes and pyocyanin pigment. Moreover, the drug combination substantially enhances the susceptibility of *P. aeruginosa* to oxidative stress, which indicates improved immunity in eliminating bacterial cells. In line with the in vitro results, the histopathological photomicrographs of mice receiving the metformin–vildagliptin combination show significant protection against *P. aeruginosa* and the alleviation of inflammation resulting from *P. aeruginosa*-induced pathogenesis. The anti-virulence activities of the metformin–vildagliptin combination can be attributed to the anti-QS activities of both metformin and vildagliptin, as both exhibit considerable affinity to QS receptors. Furthermore, the combination considerably downregulates the expression of the QS-encoding genes in *P. aeruginosa*. The effectiveness of this combination at a very low concentration encourages extending the pharmaceutical and pharmacological studies to attest to the possible clinical use of metformin–vildagliptin as an anti-virulence drug.

## Data Availability

Data are contained within the article.
